# Metabolomic response to acute resistance exercise in healthy older adults by ^1^H-NMR

**DOI:** 10.1371/journal.pone.0301037

**Published:** 2024-03-28

**Authors:** Darya Moosavi, Ivan Vuckovic, Hawley E. Kunz, Ian R. Lanza

**Affiliations:** 1 Department of Internal Medicine, Endocrine Research Unit, Division of Endocrinology, Mayo Clinic, Rochester, MN, United States of America; 2 Department of Biobehavioral Sciences, Teachers College, Columbia University, New York, NY, United States of America; 3 Department of Biochemistry and Molecular Biology, Mayo Clinic, Rochester, MN, United States of America; Dynamical Business & Science Society - DBSS International SAS, COLOMBIA

## Abstract

**Background:**

The favorable health-promoting adaptations to exercise result from cumulative responses to individual bouts of physical activity. Older adults often exhibit anabolic resistance; a phenomenon whereby the anabolic responses to exercise and nutrition are attenuated in skeletal muscle. The mechanisms contributing to age-related anabolic resistance are emerging, but our understanding of how chronological age influences responsiveness to exercise is incomplete. The objective was to determine the effects of healthy aging on peripheral blood metabolomic response to a single bout of resistance exercise and whether any metabolites in circulation are predictive of anabolic response in skeletal muscle.

**Methods:**

Thirty young (20–35 years) and 49 older (65–85 years) men and women were studied in a cross-sectional manner. Participants completed a single bout of resistance exercise consisting of eight sets of 10 repetitions of unilateral knee extension at 70% of one-repetition maximum. Blood samples were collected before exercise, immediately post exercise, and 30-, 90-, and 180-minutes into recovery. Proton nuclear magnetic resonance spectroscopy was used to profile circulating metabolites at all timepoints. Serial muscle biopsies were collected for measuring muscle protein synthesis rates.

**Results:**

Our analysis revealed that one bout of resistance exercise elicits significant changes in 26 of 33 measured plasma metabolites, reflecting alterations in several biological processes. Furthermore, 12 metabolites demonstrated significant interactions between exercise and age, including organic acids, amino acids, ketones, and keto-acids, which exhibited distinct responses to exercise in young and older adults. Pre-exercise histidine and sarcosine were negatively associated with muscle protein synthesis, as was the pre/post-exercise fold change in plasma histidine.

**Conclusions:**

This study demonstrates that while many exercise-responsive metabolites change similarly in young and older adults, several demonstrate age-dependent changes even in the absence of evidence of sarcopenia or frailty.

**Trial registration:**

**Clinical trial registry:** ClinicalTrials.gov NCT03350906.

## Introduction

Sarcopenia is an insidious process whereby muscle mass and function progressively decline over many decades [1], eventually reaching a threshold below which physical function becomes compromised and frailty and disability become more likely. This functional decline in older adults is compounded by metabolic derangements that include insulin resistance [2], ectopic lipid accumulation [3], mitochondrial dysfunction [4, 5], oxidative stress [6] and altered protein metabolism [7, 8]. Skeletal muscle is a nexus of age-related changes in metabolic health and physical function and has emerged as a target tissue for therapeutic strategies to lessen the burden of sarcopenia. Indeed, individuals who engage in lifelong physical activity are protected from many of these undesirable changes [9], supporting the notion that the functional and metabolic declines, while hallmarks of aging, are not necessarily inevitable consequences of chronological age, *per se*. From a pragmatic viewpoint, a variety of chronic conditions make it difficult or impossible for many older adults to engage in physical activity at levels sufficient to fully maintain muscle health.

Exercise reduces disease and disability in older adults, but many individuals fail to demonstrate favorable adaptations even when interventions are painstakingly controlled [10, 11]. This so-called anabolic resistance is particularly evident in older adults, who exhibit blunted anabolic responses to exercise and nutrition [12, 13], which likely contributes to sarcopenia and attenuated adaptive responses to exercise. In the quest to fully understand the factors that regulate exercise response in older adults, several potential mechanisms have been proposed including dysregulated intracellular signaling pathways [14], altered mitochondrial function [5], and chronic inflammation [15, 16]. Although substantial precedent literature is devoted to understanding how aging influences adaptive responses to exercise, there are still gaps in the current knowledge base related to detailed molecular and cellular signals that influence these adaptive responses [17]. Toward this goal, metabolomics has emerged as an analytical tool to provide a window into metabolic processes. Metabolites are the fingerprints of these processes in the form of small molecules in tissues and biological fluids such as blood and urine. Moreover, many metabolites are believed to act as signaling molecules that influence other cellular processes in endocrine or paracrine manner [18, 19]. To date, metabolomics studies of acute exercise response have mostly been conducted in response to endurance/aerobic exercise [20], with fewer investigations of acute resistance exercise [21]. In the context of aging, metabolomics studies of acute resistance exercise are scarce, but hold promise to generate new insights into the factors that regulate or attenuate anabolic response to resistance exercise in older adults. The possibility of generating molecular insights from peripheral blood sampling is particularly appealing for difficult-to-study human populations such as pediatric and older adults. The objective of this study was to determine the effects of healthy aging on peripheral blood metabolomic response to a single bout of resistance exercise and whether any circulating metabolites are predictive of anabolic response in skeletal muscle.

## Subjects and methods

### Study design

This study is a secondary analysis of data and biospecimens collected as part of a larger clinical trial (ClinicalTrials.gov identifier: NCT03350906). The metabolomics analysis was not predeclared as a primary or secondary endpoint of the parent trial and should be regarded exploratory post-hoc analyses. **S1 Table** of the provides the CONSORT Checklist for the study.

### Participants

All procedures were carried out according to the Declaration of Helsinki and were approved by the Mayo Foundation Institutional Review Board (IRB 17–004403). All participants provided written informed consent. Thirty young (27.1 [4.11] years) men and women and 49 older (71.4 [4.53] years) men and women were recruited from the southeast Minnesota area from February 2018 through December 2021. Inclusion criteria were age 20–35 years of age for the young group and 65–85 years of age for the older group. Participants were excluded if they reported diabetes or fasting plasma glucose greater than 126 mg/dL, anemia (hemoglobin less than 11 g/dL for females and less than 12 g/dL for males), active coronary artery disease or history of unstable macrovascular disease, renal failure (serum creatinine greater than 1.5 mg/dL), active liver disease (AST greater that 144 IU/L or ALT greater than 165 IU/L), history of blood clotting disorders, anticoagulant therapy, international normalized ratio (INR) greater than 2.0, substance abuse, untreated or uncontrolled hypothyroidism, pregnancy or breastfeeding. Participants were also excluded if they reported engaging in structured exercise training more than 20 minutes three times per week.

### Outpatient muscle strength testing

Knee extensor muscle strength was determined from unilateral one-repetition maximum (1-RM). Participants were familiarized with a pneumatic resistance leg extension machine (Keiser Air300, Keiser Corporation, Fresno, CA, USA), instructed in the proper range of motion, and allowed to perform a warm-up set of 10 repetitions at minimal resistance. Following the warm-up set, participants performed three sets of 5–10 repetitions at progressively increasing resistance at the discretion of the investigator and tailored based on participant’s perceived exertion. Three minutes of rest was provided between sets. Following habituation, 1-RM was determined from a series of single attempts at incremental resistance with three min of rest between attempts [22, 23]. 1-RM was defined as the maximum load that could be moved through the full range of motion with proper form.

### Inpatient acute exercise session

Within three months of completing outpatient testing, participants were admitted to the Clinical Research and Trials Unit. In advance of this visit, participants were provided with three days of weight-maintaining meals (20% protein, ~50% carbohydrate, and ~30% fat) using the Mifflin-St Jeor equation to estimate resting metabolic rate (RMR) and total daily energy expenditure from RMR and an activity factor [24]. On the evening of admission to the research unit, participants ate an evening meal at ~1800h and remained fasting except for water until completion of the study the following morning. Participants completed eight sets of 10 repetitions of unilateral knee extensions at 70% of 1-RM. Vastus lateralis muscle biopsies were performed under local anesthesia (2% lidocaine) using a modified Bergstrom needle [44] before exercise (non-exercised leg) and three hours following exercise (exercised leg). Muscle tissue was immediately blotted on sterile gauze, weighed at the bedside, and frozen in liquid nitrogen before being transferred to -80°C freezer until analysis of muscle protein synthesis rates. Blood samples were collected 30 minutes before exercise, and immediately following completion of the final set, 30-, 90-, and 180-minutes post exercise. Whole blood was placed on ice and processed immediately for plasma collection. Plasma samples were stored at -80°C until metabolomics analysis.

### Muscle protein synthesis

For muscle protein synthesis measurements, [^13^C_6_]phenylalanine was administered through a peripheral intravenous catheter at 0500h as an initial bolus dose (1mg/kg fat free mass) followed by continuous infusion (1 mg/kg fat free mass/hour). Another intravenous catheter was placed retrograde in the opposite hand, which was kept in a plexiglass box maintained at 55°C for collection of arterialized venous blood samples. Muscle fractional synthesis rates (FSR) were calculated from the increment in isotopic enrichment of [^13^C_6_]phenylalanine in the mixed muscle protein (MMP) pool over the two biopsy timepoints with muscle tissue fluid (TF) enrichment as the precursor pool as previously described [25, 26]. Frozen muscle tissue was pulverized, and TF free amino acids were extracted with 5% sulfosalicylic acid. The remaining muscle tissue was hydrolyzed overnight in 6N HCL at 110°C. The hydrolyzed muscle protein and TF samples were purified by cation exchange columns (AG 50W-X8 resin; Bio-Rad), dried, and derivatized to isobutyl esters [27]. Phenylalanine molar percent excess was determined by tandem mass spectrometry with selective ion ***monitoring*** at 222.4 > 121.6 and 226.4 > 125.6 for the m + 2 and m + 6 fragments of ***phenylalanine*** and [^13^C_6_]phenylalanine, respectively [27] and a 6-point enrichment standard curve. Muscle FSR was calculated from the following precursor-product equation:

$$FSR=\left(\frac{E_{p2}-E_{p1}}{E_{\mathrm{precursor}}\times t}\right)\times 100$$

*E*_*p2*_ and *E*_*p1*_ are the MMP-bound enrichments of [^13^C_6_] phenylalanine in serial muscle biopsies, *E*_*precursor*_ is the isotope enrichment in TF free amino acids, and *t* is the time in hours between the two biopsies.

### ^1^H-NMR metabolomics

Plasma samples were analyzed using high-resolution proton nuclear magnetic resonance (^1^H-NMR) spectroscopy according to the Bruker B.I. QUANT-PS 2.0 standard platform as previously described [28]. The NMR instrument was calibrated using a sealed reference sample provided by the manufacturer to ensure a maximal internal devation of four % or less. The technical reproducibility of abundant metabolites (e.g., glucose, alanine, glycine, lactate, pyruvate) is less than 5%, while other less abundant metabolites are 5–15% coefficient of variation. Plasma samples were thawed on ice and mixed with Bruker VERBR plasma buffer (phosphate buffer pH 7.4 containing 4.6 mM TSP-*d*_4_ (sodium 3-(trimethylsilyl) (2,2,3,3-*d*_4_) propionate and 20% of D_2_O) in 1:1 (v/v) ratio. The samples were prepared as follows: 300 μL of plasma was mixed with 300 μL of phosphate buffer, gently shaken for one minute and transferred to a five mm NMR tube. The NMR spectra were collected using a Bruker 600 MHz Avance III HD spectrometer with a BBI room temperature probe head and SampleJet auto sampler (Bruker Biospin, Billerica, MA). The sample temperature in the magnet was regulated to 310 ± 0.1 K with a BTO 2000 variable temperature unit. The 1D NMR spectra were recorded with water peak suppression using the standard 1D NOESY pulse sequence (noesygppr1d; Bruker BioSpin), acquiring 32 scans, with 98,304 data points, a spectral width of 18,029 Hz, a mixing time of 10 ms, acquisition time of 2.73 s, and a relaxation delay of four seconds. Before Fourier transformation, the line broadening of 0.3 Hz (LB = 0.3 Hz) was applied to the free induction decay. The phase and baseline corrections were performed automatically. Spectra were transferred to the Bruker Data Analysis server for automated remote analysis. Identified metabolites and their corresponding peak assignments from the 1H-NMR spectra are shown in **S1 Fig**. An example of metabolite quantitation using the Bruker IVDR method is provided for a reference plasma sample (SRM 1950) in **S2 Fig**.

### Statistical analysis

Descriptive and clinical characteristics of the study participants at baseline are provided as means and standard deviations with unpaired t-tests utilized to compare differences between young and older groups. Data were assessed for normality by Shapiro-Wilk test and histograms. To address the non-normal distribution and the dependency inherent in the repeated measures design, a Generalized Linear Mixed Model (GLMM) approach was implemented. Employing the ’lme4’ package in R, GLMMs were conducted to determine the present of significant main effects for each independent variable (age and exercise) and their interaction (age*exercise). A random intercept was included to account for variability between samples. The exercise*age interaction term was used to determine if changes in metabolite concentrations with acute exercise depended on participants’ age groups. In the absence of a significant exercise*age interaction, the main effects of exercise were used to determine if variables were different at any of the five sampling time points regardless of age. Likewise, the main effect of age was used to determine if metabolite concentrations differed by age group, regardless of exercise-induced responses to intervention. If there was a significant interaction, the Tukey’s Honest Significant Difference (HSD) test was used to compare the mean concentrations between the two groups at each sampling time point. The False Discovery Rate (FDR) method was applied to adjust p-values, thus mitigating the risk of Type I errors associated with multiple testing. P-values were compared to the level of statistical significance, which was set at α = 0.05. All results were analyzed using RStudio, version 3.4.1.

## Results

### Participant characteristics

A total of 30 young and 49 older adults were included. Young and older adults had similar height, mass, and BMI (**Table 1**). Older adults had significantly higher systolic blood pressure compared to young, but there was no difference in diastolic blood pressure (**Table 1**). Fasting plasma glucose was significantly elevated in older compared to young adults with no significant difference in fasting insulin values between age groups (**Table 1**).

**Table 1:** Demographic and clinical characteristics of young and older adults at baseline.

	Young (N = 30)		Old (N = 49)		Young vs Old
	Range min-max	Mean (SD)	Range min-max	Mean (SD)	P-value
Sex, F/M	15F-15M		26F-23M		
Age, years	20–30	27.07 (4.11)	65–84	71.37 (4.53)	<0.001*
Height, cm	154.3–185.5	171.35 (8.56)	149.6–192.05	168.74 (9.95)	0.237
Mass, kg	53.13–97.50	73.77 (11.35)	50.27–111.23	75.13 (13.45)	0.645
BMI, kg/m	19.90–28.95	24.91 (2.70)	21.45–37.20	26.28 (3.65)	0.079
Heart rate, BPM	48–92	67.52 (10.84)	46.50–87	63 (8.50)	0.042*
SBP, mm Hg	97.50–139.50	116.03 (10.10)	107–170	129.70 (13.63)	<0.001*
DBP, mm Hg	47–86	72.08 (9.13)	53–93	72.95 (9.71)	0.693
Glucose, mg/dL	72–100	86.00 (7.48)	80–121	93.47 (8.31)	<0.001*
Insulin, μIU/mL	2.9–16.4	7.37 (3.33)	2.4–29.7	7.90 (5.55)	0.60

*Note*: M; Male, F; Female, BMI; body mass index, SBP; systolic blood pressure, DBP; diastolic blood pressure

*; statistically significant at the .05 level. Data are shown as mean (SD).

### Pre-exercise plasma metabolomics

**Fig 1:**
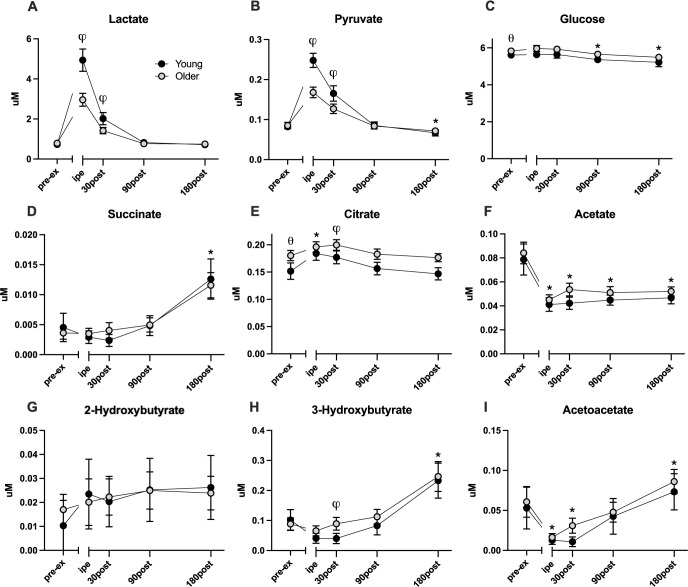
Changes in plasma carbohydrate, TCA cycle intermediates, and ketone body metabolites in young (*n* = 30) and older (*n* = 49) adults pre and post resistance exercise. Values are shown as mean and 95% CIs of non-transformed data, with (θ) denoting significant (p-value < 0.05) difference between young and older groups at baseline, (*) representing significant effect of time from baseline, (φ) representing age and time interaction, found by LMM. Pre-ex: pre-exercise, IPE: immediately post exercise.

**Fig 2:**
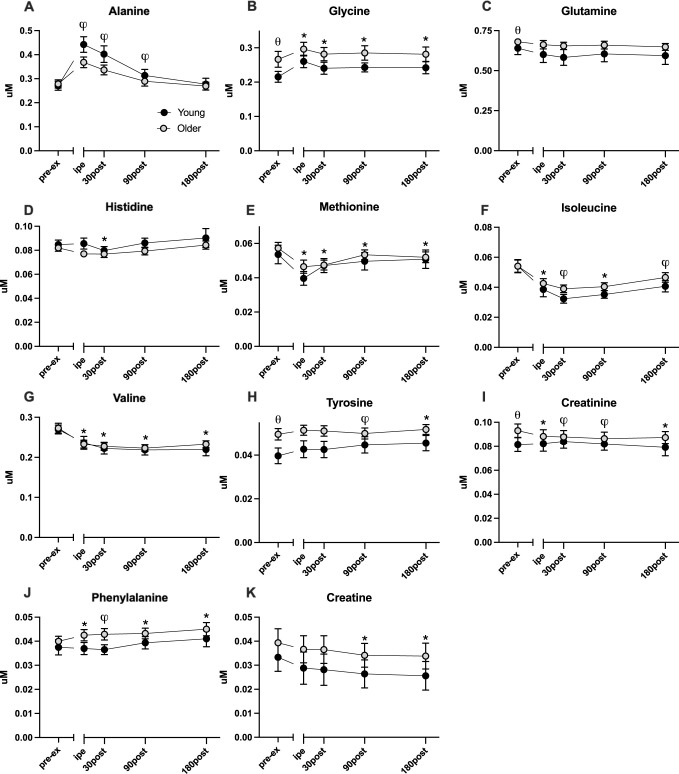
Changes in plasma glucogenic and biogenic amines in young (*n* = 30) and older (*n* = 49) adults pre and post resistance exercise. Values are shown as mean and 95% CIs of non-transformed data, with (θ) denoting significant (p-value < 0.05) difference between young and older groups at baseline, (*) representing significant effect of time from baseline, (φ) representing age and time interaction, found by LMM. Pre-ex: pre-exercise, IPE: immediately post exercise.

**Fig 3:**
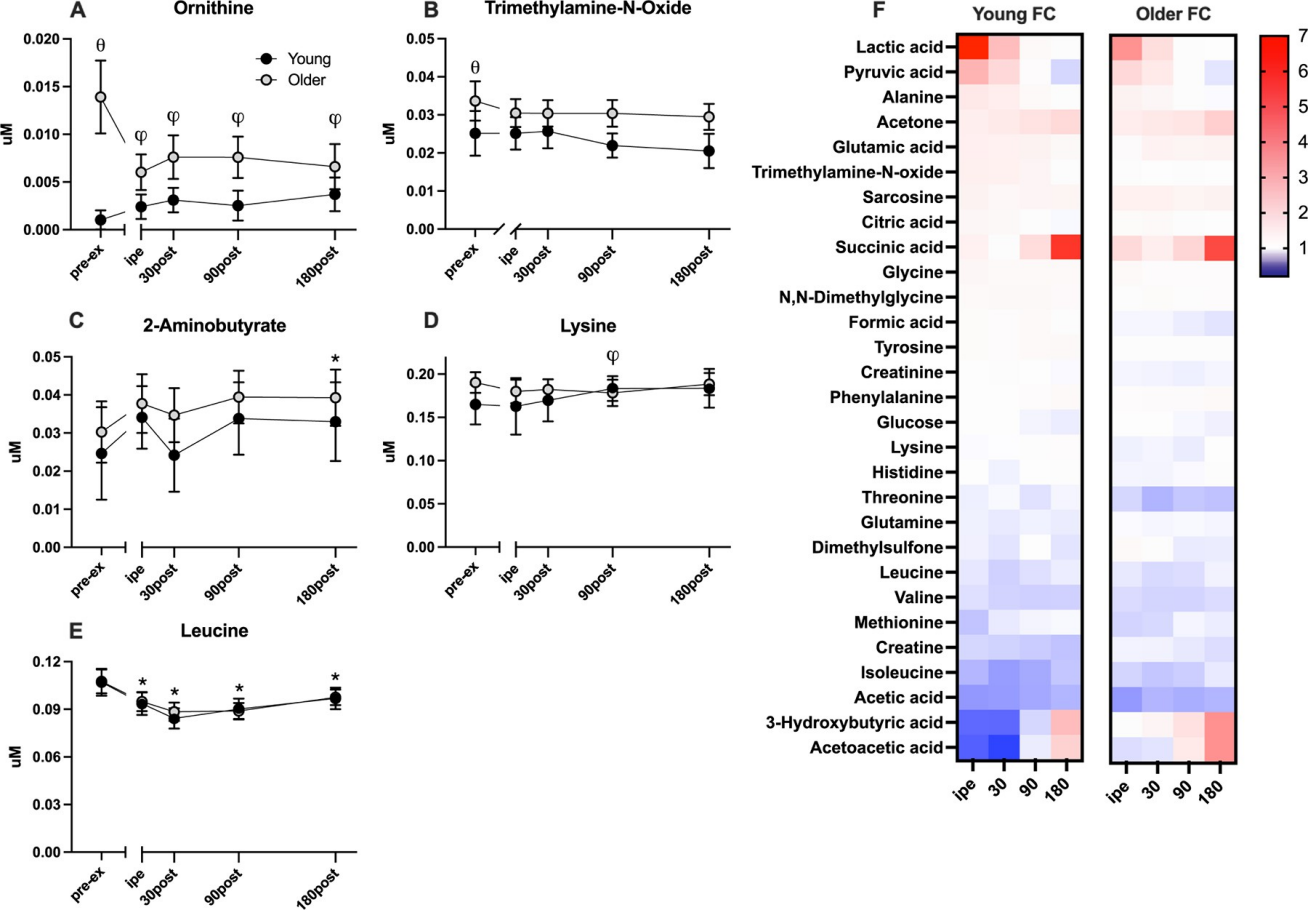
Changes in other amino acids and metabolites (A-E), and fold-change (F) pre and post resistance exercise in young (*n* = 30) and older (*n* = 49) adults. Values are shown as mean and 95% CIs of non-transformed data, with (θ) denoting significant (p-value < 0.05) difference between young and older groups at baseline, (*) representing significant effect of time from baseline, (φ) representing age and time interaction, found by LMM. Pre-ex: pre-exercise, IPE: immediately post exercise.

Plasma ^1^H-NMR metabolomics in baseline pre-exercise plasma samples (**Figs 1–3**) revealed elevated plasma glucose (P = 0.043) concentration in older compared to younger adults. Glycine (P<0.0001), glutamine (P = 0.042), tyrosine (P<0.0001), creatinine (P = 0.034), ornithine (P<0.0001), trimethylamine N-oxide (P = 0.04), and citrate (P<0.0001) were also significantly elevated in plasma from older compared to young adults at baseline.

### Metabolomic response to acute resistance exercise

Of the 33 plasma metabolites detected and quantitated by ^1^H-NMR, 26 demonstrated time-dependent changes following acute resistance exercise. The acute bout of single-leg RE elicited increased plasma organic acids (lactate, pyruvate, p< 0.001) (**Fig 1A and 1B**) and decreased ketones (acetoacetate, acetate, p < 0.001) (**Fig 1H and 1I**) and several amino acids (methionine, isoleucine, valine, ornithine, p < 0.001) (**Fig 2E–2G**) in the immediate post-exercise time points (0 and 30 minutes after exercise). In contrast, the amino acids alanine and glycine (**Fig 2A, 2B**) exhibited acute increases (p < 0.001) immediately following exercise. Other plasma metabolites demonstrated more latent changes following exercise that emerged in the later recovery period. These metabolites include succinate (**Fig 1D**), 3-hydroxybutyrate (**Fig 1H**), and acetoacetate (**Fig 1I**), which were significantly elevated at 180 minutes of recovery (p < 0.001). On the other hand, glucose (**Fig 1C**), acetate (**Fig 1F**), valine (**Fig 2G**), and isoleucine (**Fig 2F**) remained below the basal levels at 180 minutes of recovery.

Of the metabolites that were responsive to acute single-leg resistance exercise, 12 demonstrated significant exercise*age interactions, including lactate (**Fig 1A**), pyruvate (**Fig 1B**), 3-hydroxybutyrate (**Fig 1H**), acetoacetate (**Fig 1I**), alanine (**Fig 2A**), isoleucine (**Fig 2F),** tyrosine (**Fig 2H**), creatinine (**Fig 2I**), phenylalanine (**Fig 2J**), ornithine **(Fig 3A),** lysine **(Fig 3D)**. Notable trends that did not reach statistical significance included glutamine (**Fig 2C;** P = 0.055) and citrate (**Fig 1E;** P = 0.086). Plasma lactate (**Fig 1A**), pyruvate (**Fig 1B**), and alanine (**Fig 2A**) concentrations were acutely elevated immediately following exercise and returned to baseline at the 90-minute timepoint with older adults demonstrating smaller magnitude changes compared to young. Plasma ketones 3-hydroxybutyrate (**Fig 1H**) and acetoacetate (**Fig 1I**) acutely decreased in young and older adults following exercise but with greater magnitude changes evident in young compared to older. The amino acids methionine (**Fig 2E**), and isoleucine (**Fig 2F**) exhibited acute decreases immediately post-exercise that tended to be more apparent in young compared to older adults though this did not reach statistical significance.

### Associations between plasma metabolites and muscle protein synthesis

**Fig 4:**
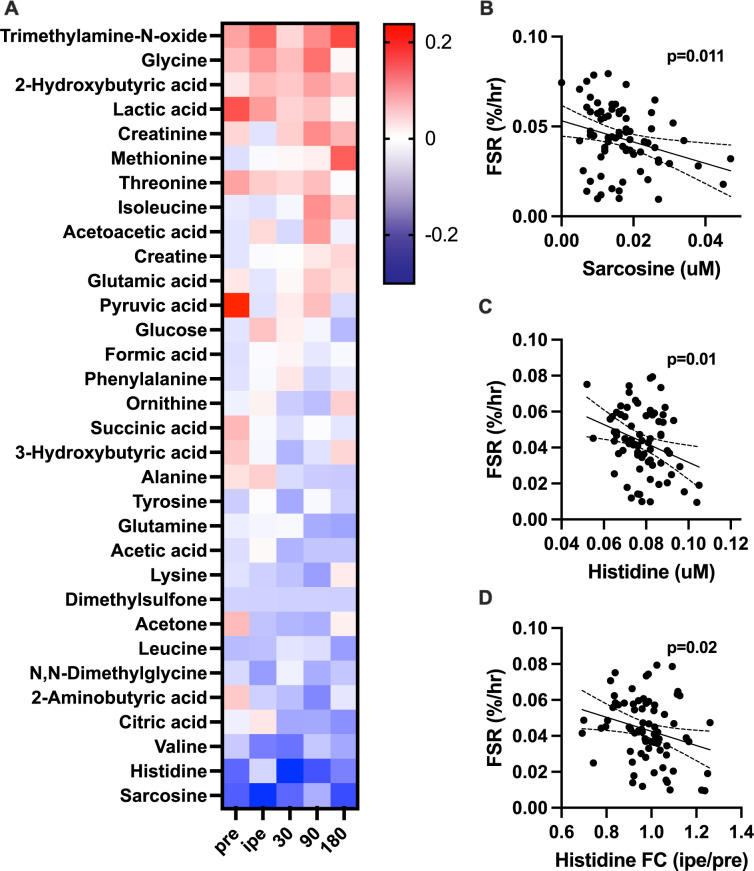
Correlation between muscle protein synthesis rates and plasma metabolite levels. The heatmap in panel A provides the correlation coefficients between muscle fractional synthesis rate (FSR) and metabolite concentrations at each timepoint. Of these, sarcosine (B) and histidine (C) concentrations at rest were significantly negatively associated with FSR. There was also a significant negative association between FSR and the fold change (FC) in plasma histidine from pre-exercise to immediate post-exercise (D). Red color represents positive Pearson correlation coefficients while blue color represents negative correlation. Dotted lines represent 95% confidence intervals for regression analyses in panels B, C, and D.

As an exploratory exercise, we examined associations between circulating metabolites and muscle protein synthesis (**Fig 4A**). Pre-exercise plasma lactate and pyruvate levels were positively associated with muscle protein synthesis, although the relationships did not reach statistical significance (P<0.10). Pre-exercise histidine **(Fig 4C)** and sarcosine **(Fig 4B)** were significantly negatively associated with muscle protein synthesis, as was the pre/post-exercise fold change in plasma histidine **(Fig 4D)**.

## Discussion

This study employed semi-quantitative NMR-based plasma metabolite profiling to compare the acute response to resistance exercise in young and older adults. We demonstrate that a single bout of unilateral resistance exercise induces significant shifts in circulating metabolites, including organic acids, ketones, and amino acids. Moreover, we identified 12 metabolites that were influenced by acute resistance exercise in a manner that was dependent on age. These metabolites included lactate, pyruvate, alanine, 3-hydroxybutyrate, ornithine, tyrosine, formate, lysine, glycerol, isoleucine, creatinine, and phenylalanine. We were interested in determining if any circulating exercise-responsive metabolites were predictive of anabolic response to exercise, measured from the rate of muscle protein synthesis. We found that muscle protein synthesis was positively associated with pre-exercise plasma lactate and pyruvate and negatively associated with histidine and sarcosine. Furthermore, the fold change in plasma histidine was negatively associated with muscle protein synthesis. Altogether these results highlight key circulating metabolites that are responsive to acute resistance exercise, including those that respond distinctly in young and older adults, and those that are associated with anabolic response.

Fasting pre-exercise levels of glucose, citrate, creatinine, and several amino acids (glycine, glutamine, tyrosine, ornithine) were elevated in older adults compared with the younger group. These findings harmonize with precedent literature that also document elevated plasma levels of these analytes in older adults [28–31]. While it is tempting to ascribe these systemic metabolite changes with age to known age-related changes to mitochondrial function [4, 5] and muscle protein metabolism [7, 8], the tissues of origin and precise metabolic processes that contribute to this metabolite fingerprint are uncertain. Our main objective was to assess the time-dependent changes in plasma metabolites in response to acute resistance exercise in young and older adults. The majority of the exercise-responsive metabolites changed similarly in young and older, although several exhibited age-dependent changes in response to exercise. Plasma glucose levels increased immediately following RE and declined below the basal levels 180 minutes post-exercise. This observation can be attributed to the enhanced glucose uptake in skeletal muscle that continues for hours following exercise onset [32]. Our finding closely mirrored previously reported changes in which the plasma concentrations of glucose were reduced following RE in healthy [33], and diabetic individuals [34], and within 180 minutes of recovery [35]. Due to the accelerated carbohydrate utilization during RE, lactate, and pyruvate were markedly elevated immediately after exercise. Despite the early dogma of lactate as a dead-end metabolic byproduct, there is now convincing evidence that lactate production occurs continuously at rest and during exercise in fully oxygenated conditions [36–38]. Pyruvate and lactate concentrations followed a steady decreasing trend and reached the basal levels within 90 minutes of recovery. Pyruvate significantly dropped below the baseline values at 180 minutes post-RE. Our observation is in agreement with prior findings showing that blood pyruvate and lactate decline within an hour after RE [35, 39, 40]. TCA cycle intermediates are known to be increased in blood following endurance and resistance exercise, [20, 21, 39, 41–43]. Likewise, we find citrate increased immediately and 30 minutes after exercise and gradually returned to the basal levels 90 minutes into the recovery time in both young and older groups. Succinate, another notable TCA metabolite involved in muscle adaptation and remodeling [44], remained elevated 180 minutes post exercise in-line with prior reports [20, 39, 40]. Acetate, which is utilized by the TCA cycle to synthesize nicotinamide adenine nucleotide (NADH) and ATP, declined significantly following exercise and remained below basal levels at 180 minutes.

Plasma amino acids were substantially influenced by a single bout of resistance exercise. The branched-chain amino acids (BCAA) isoleucine, leucine, and valine were reduced immediately post-exercise with gradual recovery over 3 hours. A prior study of young participants reported that BCAAs decreased below basal levels as late as 60 minutes post-RE [39]. Our results show that this pattern is maintained in healthy older adults. BCAAs are relevant to exercise response as they are a carbon source for the TCA cycle [45, 46] and act as precursors for protein synthesis in the muscle [45]. Leucine is a particularly important regulator of muscle protein anabolism by activating the mTORC1 pathway [47]. Inasmuch, the observed time course of BCAA concentrations following a bout of RE are consistent with prior observations [30, 35, 39], but importantly we show that they are similar in young and older adults. In contrast to BCAAs, we observed a marked rise in alanine concentrations immediately after RE which was more pronounced in young compared to older adults. Alanine is a glucogenic amino acid that is synthesized in skeletal muscle through transamination of glutamate and released into circulation during exercise [48]. Its role as an ammonia carrier and gluconeogenic substrate makes it an important factor in the metabolic demands of resistance exercise.

Several ketone bodies including acetoacetate and 3-hydroxybutyrate demonstrated an immediate decrease after resistance exercise, followed by a later rise above pre-exercise values at 180 minutes of recovery. Ketone bodies are fuel substrates for brain and muscle under conditions such as fasting or prolonged exercise where carbohydrate availability may be limited. In our study, participants were fasted overnight and throughout exercise and the three hours of recovery. The time course of acetoacetate levels aligns with previously reported reductions in plasma acetoacetate levels in response to acute RE [20, 49]. Others have shown that 3-hydroxybutyrate and acetoacetate are released into the blood after exercise [20, 50, 51]. Berton et al. 2017 showed that 3-hydroxybutyrate and 2-hydroxybutyrate increased immediately after resistance exercise in young non-fasting individuals [39]. Previous studies have demonstrated that 3-hydroxybutyrate can regulate adaptation mechanisms in skeletal muscle through positive effects on protein synthesis in human skeletal muscle [51, 52], so the rise in 3-hydroxybutyrate during exercise likely represents a beneficial adaptive response.

While many exercise-responsive metabolites exhibited similar patterns in young and older adults, there were several metabolites that were responsive to resistance exercise in an age-dependent manner. The magnitude of exercise-induced response in metabolites associated with glycolysis and the TCA cycle, such as lactate and pyruvate **(Fig 1A and 1B),** were significantly lower in older adults compared with the younger group immediately and 30 minutes following the intervention. A similar age-related attenuation in the exercise-induced rise of alanine **(Fig 2A)** was observed, which goes together with pyruvate. Ornithine **(Fig 3A)** response in older adults was unique because, despite elevated levels at rest, ornithine was markedly lowered after exercise and remained below the basal level after 180 minutes of recovery. In contrast, ornithine concentrations in younger individuals increased immediately after exercise and remained above the baseline value throughout the three-hour recovery period. Among ketone bodies, 3-hydroxybutyrate **(Fig 1H)** exhibited different exercise responses between the young and older groups at 30 and 90 minutes into the recovery time, while acetoacetate **(Fig 1I)** concentrations exhibited a marked interaction between age and exercise response only at the 30-minute recovery timepoint. The concentration of plasma ketone bodies reflects the balance between production in liver and peripheral utilization as a fuel source. Since participants in this study remained fasting during the postexercise period, the rise in plasma ketones during this period is consistent with a glucose-sparing strategy. Although young and older adults demonstrated post-exercise ketosis, the rise was delayed in young compared to older adults, which may reflect greater peripheral utilization of ketone bodies in younger adults or differences in hepatic ketone production in young and older adults.

Circulating metabolites not only provide a window into cellular processes but may themselves influence processes through potential paracrine or endocrine influence. Inasmuch, we sought to explore the associations between skeletal muscle protein synthesis following exercise and the static levels and dynamic metabolite responses to acute exercise. The analysis revealed positive associations between muscle protein synthesis rate and plasma lactate and pyruvate concentrations at rest but not at any post-exercise time points. While these preliminary associations do not indicate causality, there is evidence that lactate may act as a signaling molecule that stimulates insulin production and activates the mTORC1 pathway, which is a crucial regulator of muscle protein synthesis [53, 54]. Histidine and sarcosine emerged as metabolites negatively associated with muscle protein synthesis at rest **(Fig 4B and 4C)**. The pre/post-exercise fold change in plasma histidine demonstrated a negative correlation with muscle protein synthesis rate. This is an unexpected finding since histidine is an essential proteinogenic amino acid required to synthesize proteins [55]. Essential amino acids activate the mTORC1 signaling pathway, the primary regulator of anabolic actions in the muscle cells, promoting muscle protein synthesis [56]. Histidine also contributes to muscle growth by producing histamine, which regulates muscle microcirculation and persistent vasodilation after exercise [57]. Additionally, histidine can act as a precursor for carnosine, a molecule that acts as an intracellular buffer, antioxidant, and free radical scavenger [55]. Studies have identified a negative association between histidine, inflammation and oxidative stress in obese individuals [57, 58]. Interestingly, in the present study, young and older individuals exhibited distinct exercise responses in Histidine levels, although they did not reach statistical significance. Following RE, histidine levels decreased in the elderly and increased in the young group. It is possible that high levels of histidine and sarcosine could inhibit the uptake of other essential amino acids in muscle cells or interfere with the function of enzymes necessary for muscle protein synthesis, although more targeted interrogation is needed to fully understand the specific mechanisms underlying the relationship between histidine, sarcosine, and muscle protein synthesis.

It is important to recognize several limitations of this work. This study evaluated a single mode of exercise (resistance) in one muscle group. The results cannot be generalized to other modes of exercise or training responses. Another key consideration is that plasma metabolites originate from many different tissues and organs, making it difficult to ascribe changes in circulating concentrations to any particular source. Another limitation of the study is that NMR spectroscopy, despite its specificity and reproducibility, is an analytical platform that has low sensitivity for low abundance molecules. Inasmuch, this platform covers a narrow biochemical space that likely excludes biologically relevant analytes that change with aging and exercise. Finally, the observational and cross-sectional nature of this work cannot definitively or mechanistically link metabolite changes with any biological processes.

In conclusion, our study highlights circulating metabolites that are acutely responsive to a single bout of resistance exercise (organic acids, ketones, amino acids), and several that exhibit age-dependent responses (lactate, pyruvate, alanine, ornithine, acetoacetate, 3-hydroxybutyrate). Of these metabolites, resting plasma lactate and pyruvate were positively associated with the anabolic response to exercise while histidine and sarcosine were negatively associated. Altogether this study confirms that many of the metabolites know to be acutely responsive to resistance exercise are also evident in healthy older adults. Nevertheless, a smaller number exhibit responses that are attenuated in older adults and are predictive of anabolic response to exercise. Here we studied relatively healthy older adults free from many common chronic conditions that accompany aging and likely influence outcomes. Inasmuch, caution should be used in generalizing the results from this study to the overall population of individuals over the age of 65 years.

## Supporting information

S1 Checklist *PLOS ONE* clinical studies checklist. (DOCX)

S1 Table CONSORT checklist. (DOC)

S1 Fig ^1^H NMR spectrum of NIST SRM 1950 plasma sample. (PDF)

S2 Fig Bruker IVDR analysis report. (PDF)

## Abbreviations

NMR: Nuclear Magnetic Resonance Spectroscopy
RE: Resistance exercise
BCAA: Branched-chain amino acids
