# Supplementary material for: Metabolomic response to acute resistance exercise in healthy older adults by 1H-NMR
Source: PLoS One. 2024 Mar 28;19(3):e0301037. doi: 10.1371/journal.pone.0301037 (PMC10977811; doi:10.1371/journal.pone.0301037)
Supplement: S1 Fig — (PDF) [file pone.0301037.s003.pdf]

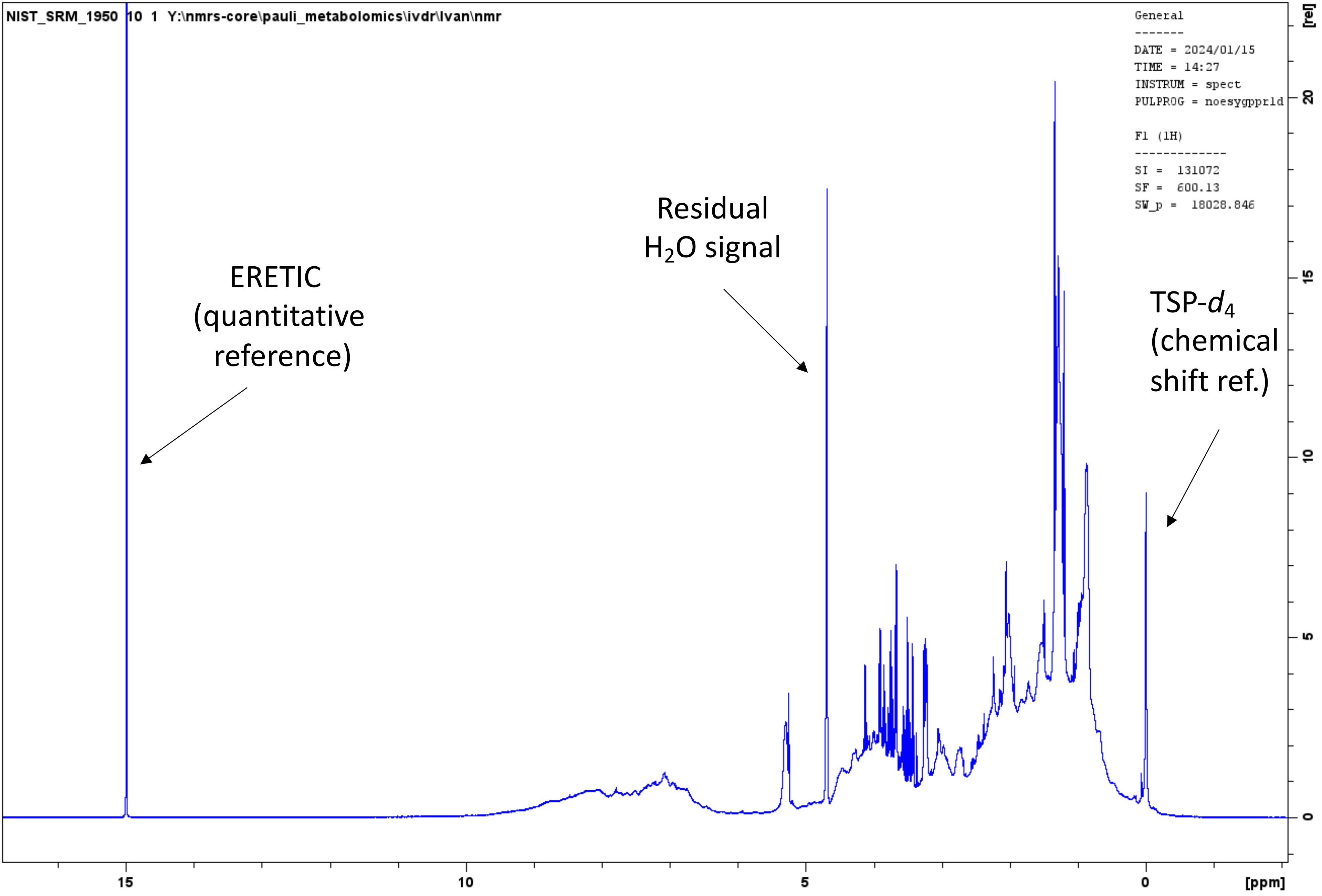

^{1}H NMR spectrum (1D NOESY with presaturation) of NIST SRM 1950 plasma sample

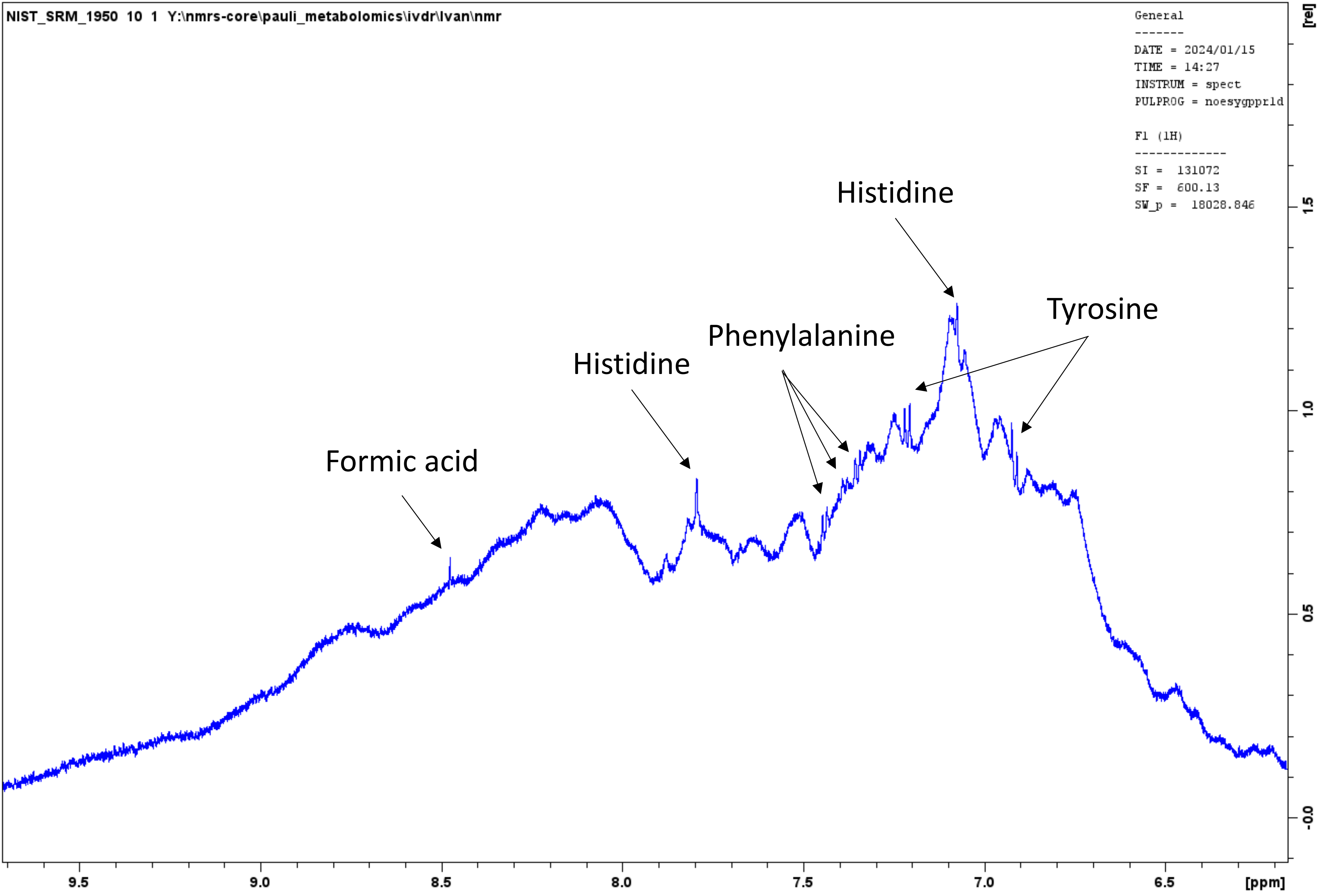

^{1}H NMR spectrum of NIST SRM 1950 plasma sample (expansion 1)

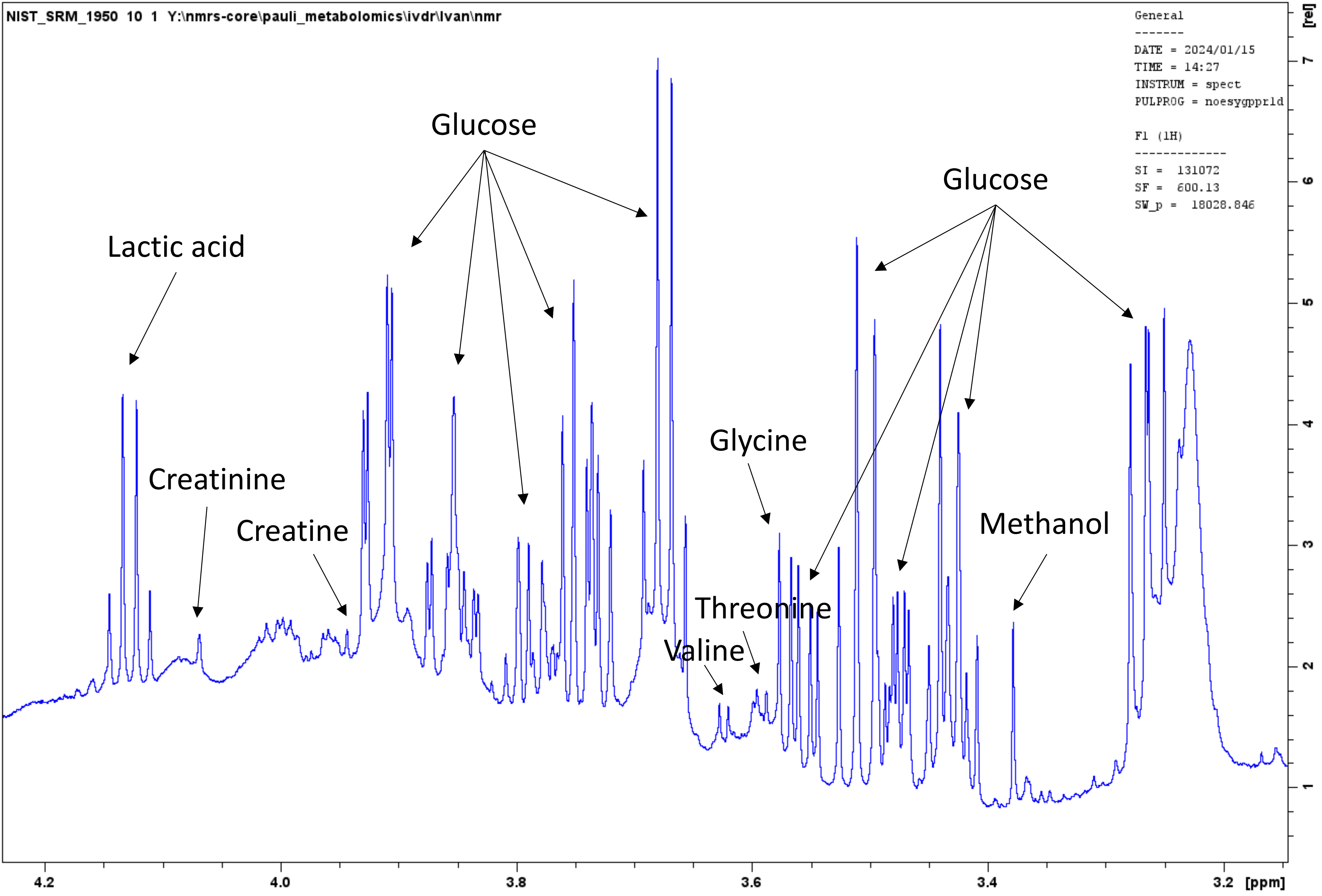

^{1}H NMR spectrum of NIST SRM 1950 plasma sample (expansion 2)

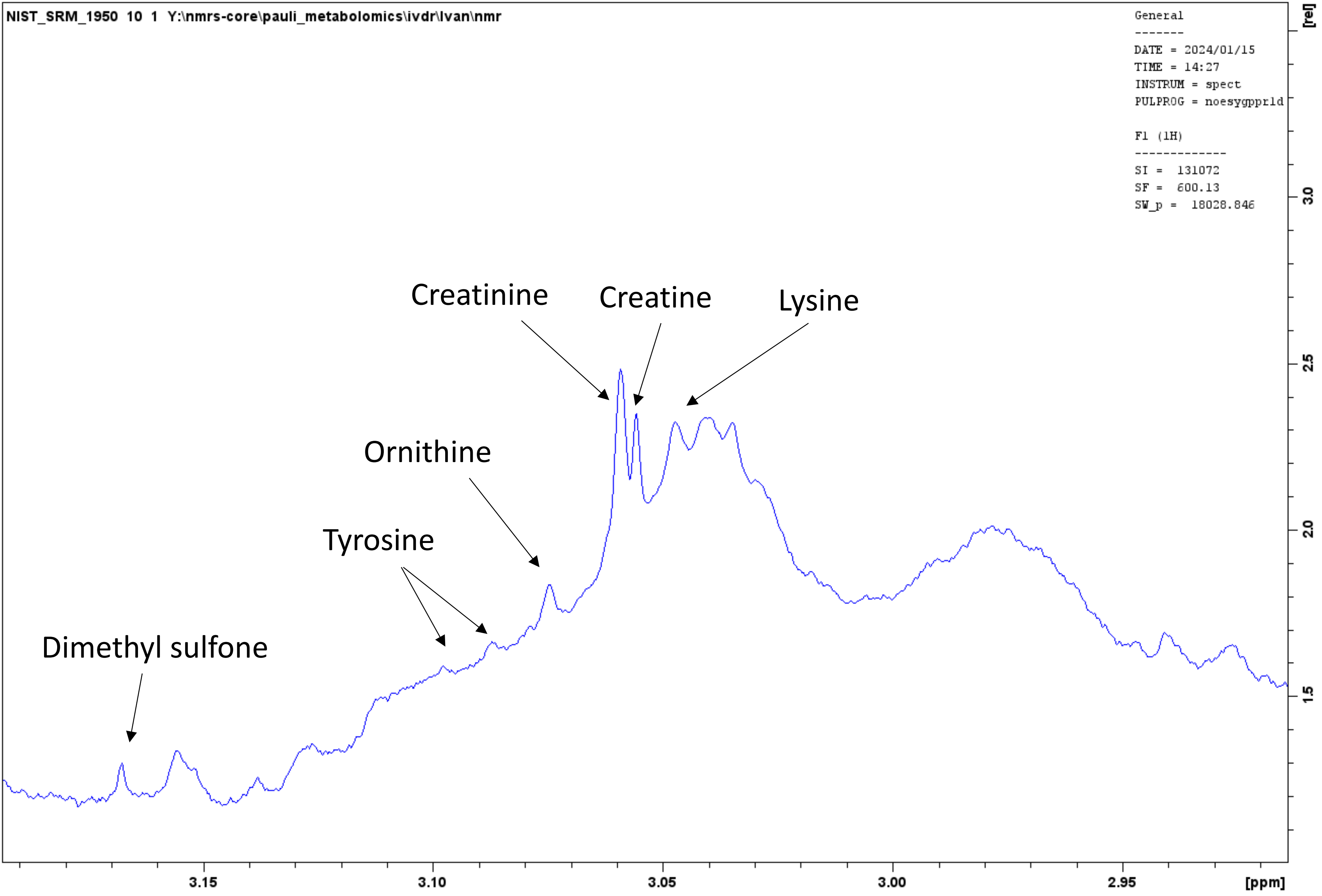

^{1}H NMR spectrum of NIST SRM 1950 plasma sample (expansion 3)

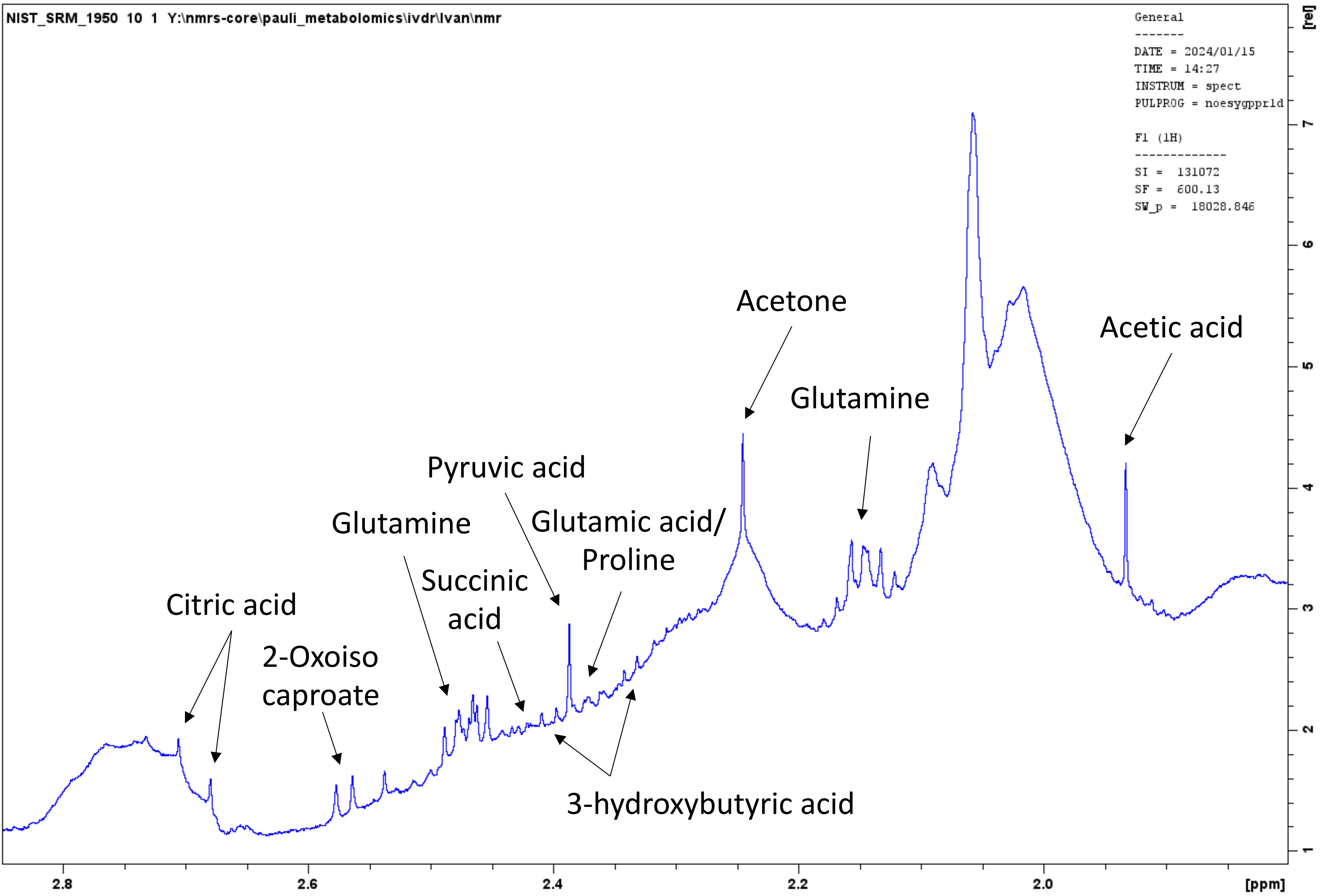

^1H NMR spectrum of NIST SRM 1950 plasma sample (expansion 4)

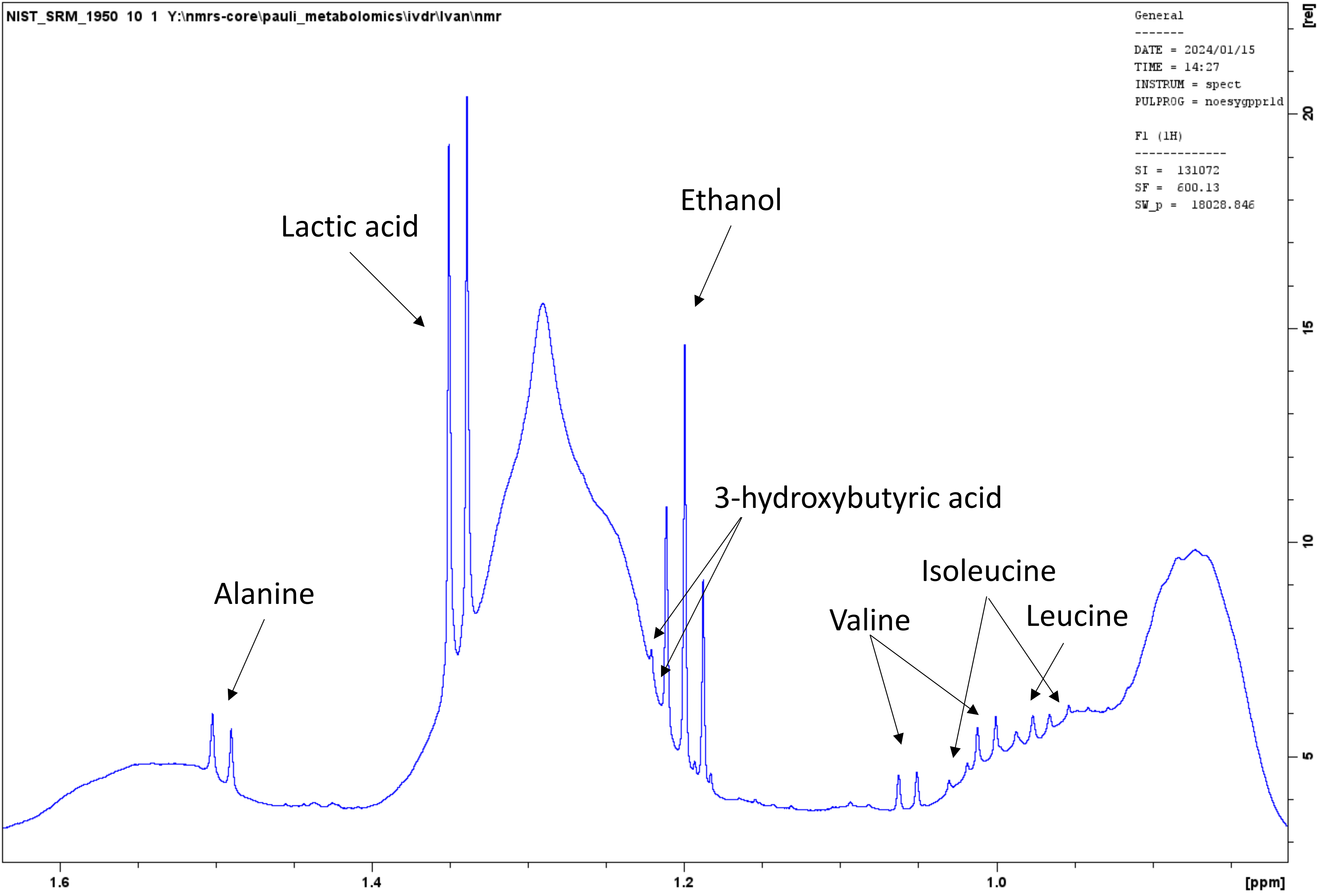

^{1}H NMR spectrum of NIST SRM 1950 plasma sample (expansion 5)
