# Supplementary material for: Metabolomic response to acute resistance exercise in healthy older adults by 1H-NMR
Source: PLoS One. 2024 Mar 28;19(3):e0301037. doi: 10.1371/journal.pone.0301037 (PMC10977811; doi:10.1371/journal.pone.0301037)
Supplement: S2 Fig — (PDF) [file pone.0301037.s004.pdf]

Analysis Report

Bruker IVDr Quantification in Plasma/Serum B.I.Quant-PS™

Sample ID: NIST_SRM_1950_expno10.100000.11r

Measuring Date:	15-Jan-2024 20:27:41
Reporting Date:	15-Jan-2024 21:31:25, 7 page(s), Version 2.0.0
Quantification Method Version:	Quant-PS 2.0.0

Disclaimer

RESEARCH USE ONLY: This is no clinical diagnostic analysis report. Must not be used for clinical (medical or IVD) diagnosis or for patient management! Additional concentration range information (95% range) provided numerically or graphically in this report must not be used for clinical diagnostic interpretation.

Application of B.I.QuantPS 2.0 requires use of Bruker's B.I.Methods SOP for plasma and serum.

Summary

The spectroscopic fingerprint of the sample is consistent with a serum or a heparin plasma profile. The following metabolites were found with concentrations outside the 95% range of Bruker Quant-PS 2.0.0 plasma/serum metabolite concentration database:

Alcohols and derivatives: *Ethanol (2.2 mmol/L),*
Carboxylic acids: *Acetic acid (0.09 mmol/L),*
Keto acids and derivatives: *3-Hydroxybutyric acid (0.29 mmol/L), Acetone (0.07 mmol/L), Pyruvic acid (0.09 mmol/L).*

Further detailed information is provided on the following pages.

Geschäftsführer: Dr. Falko Busse | Bernd Gleixner | Patrick Minhorst

Rudolf-Plank-Str. 23
76275 Ettlingen, Germany
Tel. : +49(0) 7243 7695-555
Clinical.AIC@bruker.com
www.bruker.com

Deutsche Bank AG Karlsruhe
Konto 13 15 16 · BLZ 660 700 04
IBAN: DE87 6607 0004 0013 1516 00
SWIFT-BIC: DEUTDESM660

USt-Ident.-Nr DE 143 239 759
WEEE-Reg.-Nr. DE 43 181 702
Steuer-Nr. 31190/39205
Handelsregister Mannheim HRB 10 23 68
Sitz der Gesellschaft: 76275 Ettlingen

Contents

1 Alcohols and derivatives **3**

2 Amines and derivatives **3**

3 Amino acids and derivatives **3**

4 Carboxylic acids **4**

5 Essential nutrient **4**

6 Keto acids and derivatives **4**

7 Sugars and derivatives **4**

8 Sulfones **5**

9 Technical additives **5**

10 Explanations **6**

10.1 How to read the result 6

10.1.1 Result parameters 6

10.1.2 Different fit situations 7

10.2 Detailed definitions 7

1 Alcohols and derivatives

Compound	Conc. mmol/L	LOD mmol/L	r mmol/L	ρ %	Δ mmol/L	95% Range mmol/L	Graphics (*)
Ethanol	2.2	0.10	2.160	100 ●	0.082	⩽ 0.82	

(*) Gray horizontal boxes represent 95% concentration range, black vertical lines represent sample value.

2 Amines and derivatives

Compound	Conc. mmol/L	LOD mmol/L	r mmol/L	ρ %	Δ mmol/L	95% Range mmol/L	Graphics (*)
Trimethylamine-N-oxide	< 0.08	0.08	0.015	98 ●	0.038	⩽ 0.08	

(*) Gray horizontal boxes represent 95% concentration range, black vertical lines represent sample value.

3 Amino acids and derivatives

Compound	Conc. mmol/L	LOD mmol/L	r mmol/L	ρ %	Δ mmol/L	95% Range mmol/L	Graphics (*)
2-Aminobutyric acid	< 0.05	0.05	0.000	0 ○	0.711	⩽ 0.10	
Alanine	0.30	0.02	0.304	100 ●	0.008	0.29 - 0.64	
Asparagine	< 0.05	0.05	0.000	0 ○	3.666	⩽ 0.08	
Creatine	0.02	0.01	0.022	99 ●	0.003	⩽ 0.07	
Creatinine	0.08	0.01	0.077	99 ●	0.003	0.06 - 0.14	
Glutamic acid	0.06	0.05	0.060	39 ○	0.047	⩽ 0.24	
Glutamine	0.55	0.02	0.551	99 ●	0.019	0.30 - 0.83	
Glycine	0.27	0.01	0.273	100 ●	0.006	0.17 - 0.44	
Histidine	0.07	0.02	0.072	99 ●	0.002	0.07 - 0.16	
Isoleucine	0.05	0.03	0.054	99 ●	0.004	0.03 - 0.11	
Leucine	0.08	0.01	0.083	97 ●	0.008	0.07 - 0.20	
Lysine	0.14	0.04	0.145	66 ○	0.056	⩽ 0.29	
Methionine	0.06	0.05	0.062	95 ●	0.007	0.05 - 0.13	
N,N-Dimethylglycine	< 0.01	0.01	0.003	75 ○	0.001	⩽ 0.01	
Ornithine	< 0.02	0.02	0.000	0 ○	1.820	⩽ 0.16	
Phenylalanine	0.05	0.03	0.047	97 ●	0.003	⩽ 0.07	
Proline	0.28	0.05	0.284	42 ○	0.243	⩽ 0.59	
Sarcosine	< 0.01	0.01	0.000	0 ○	0.089	⩽ 0.01	
Threonine	0.05	0.04	0.052	63 ○	0.064	⩽ 0.24	
Tyrosine	0.04	0.03	0.045	98 ●	0.003	⩽ 0.08	
Valine	0.17	0.03	0.171	100 ●	0.002	0.15 - 0.35	

(*) Gray horizontal boxes represent 95% concentration range, black vertical lines represent sample value.

4 Carboxylic acids

Compound	Conc. mmol/L	LOD mmol/L	r mmol/L	ρ %	Δ mmol/L	95% Range mmol/L	Graphics (*)
2-Hydroxybutyric acid	< 0.15	0.15	0.000	0 ○	1.407	⩽ 0.17	
Acetic acid	0.09	0.01	0.088	100 ●	0.002	⩽ 0.06	
Citric acid	0.12	0.03	0.119	97 ●	0.017	⩽ 0.21	
Formic acid	0.03	0.02	0.026	95 ●	0.001	⩽ 0.03	
Lactic acid	2.3	0.03	2.272	100 ●	0.049	2.23 - 7.14	
Succinic acid	< 0.01	0.01	0.002	98 ●	0.000	⩽ 0.01	

(*) Gray horizontal boxes represent 95% concentration range, black vertical lines represent sample value.

5 Essential nutrient

Compound	Conc. mmol/L	LOD mmol/L	r mmol/L	ρ %	Δ mmol/L	95% Range mmol/L	Graphics (*)
Choline	< 0.05	0.05	0.000	0 ○	0.099	⩽ 0.06	

(*) Gray horizontal boxes represent 95% concentration range, black vertical lines represent sample value.

6 Keto acids and derivatives

Compound	Conc. mmol/L	LOD mmol/L	r mmol/L	ρ %	Δ mmol/L	95% Range mmol/L	Graphics (*)
2-Oxoglutaric acid	< 0.02	0.02	0.000	0 ○	1.715	⩽ 0.02	
3-Hydroxybutyric acid	0.29	0.02	0.290	69 ○	0.184	⩽ 0.26	
Acetoacetic acid	< 0.01	0.01	0.006	98 ●	0.000	⩽ 0.02	
Acetone	0.07	0.01	0.071	100 ●	0.001	⩽ 0.06	
Pyruvic acid	0.09	0.03	0.094	100 ●	0.001	⩽ 0.07	

(*) Gray horizontal boxes represent 95% concentration range, black vertical lines represent sample value.

7 Sugars and derivatives

Compound	Conc. mmol/L	LOD mmol/L	r mmol/L	ρ %	Δ mmol/L	95% Range mmol/L	Graphics (*)
D-Galactose	< 0.11	0.11	0.000	0 ○	1.511	⩽ 0.11	
Glucose	4.6	0.54	4.635	100 ●	0.011	1.73 - 6.08	
Glycerol	< 0.08	0.08	0.000	0 ○	1.032	⩽ 0.44	

(*) Gray horizontal boxes represent 95% concentration range, black vertical lines represent sample value.

8 Sulfones

Compound	Conc. mmol/L	LOD mmol/L	r mmol/L	ρ %	Δ mmol/L	95% Range mmol/L	Graphics (*)
Dimethylsulfone	< 0.01	0.01	0.006	100	0.000	⩽ 0.02	

(*) Gray horizontal boxes represent 95% concentration range, black vertical lines represent sample value.

9 Technical additives

Compound	Conc. mmol/L	LOD mmol/L	r mmol/L	ρ %	Δ mmol/L	95% Range mmol/L	Graphics (*)
Ca-EDTA	< 0.50	0.50	0.022	100	0.001	⩽ 0.50	
K-EDTA	< 0.50	0.50	0.014	99	0.001	⩽ 0.50	

(*) Gray horizontal boxes represent 95% concentration range, black vertical lines represent sample value.

10 Explanations

This section contains the definition of the parameters used above. In the section 10.1 a short manual, how to interpret the results, is presented. The section 10.2 contains the exact definitions of the parameters $\mathbf{r}$, ρ and $\boldsymbol{\Delta}$.

10.1 How to read the result

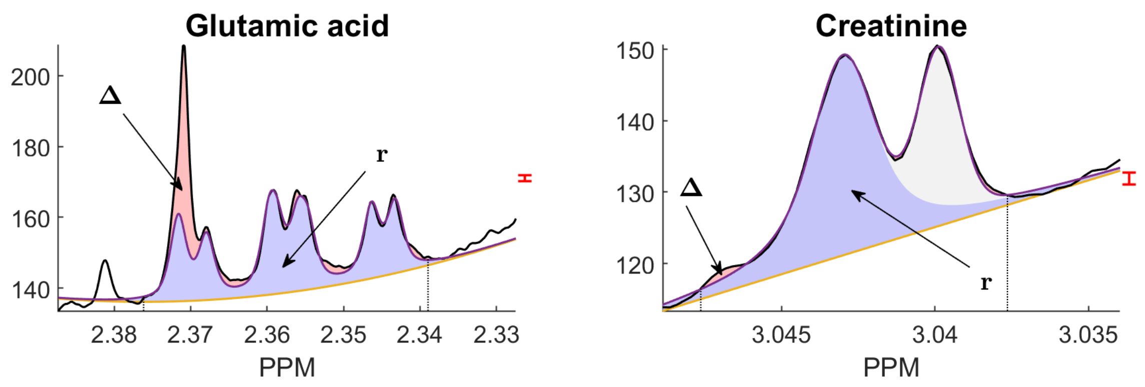

(a) Single signal fitting.

(b) Simultaneous fitting of two signals.

Figure 1: Examples of fitting.

In the figure 1(a), the black line, the blue line and the yellow line represent the original spectrum, the calculated signal fit and its baseline, respectively. Additionally, the red bar on the right side of the plot, indicates the 95% noise range.

The blue area relates to the metabolite concentration to be determined and the red area represents a residue.

In case of the signal overlap a different approach is used: two or more overlapping signals are being fitted simultaneously. The most iconic example of such signals are the ones generated by CH_3 groups of Creatinine and Creatine. In such a case, the blue line and the grey area relate the sum of all fitted signals. The blue area corresponds to the concentration of the metabolite of interest (cf. figure 1(b)).

10.1.1 Result parameters

a) **Conc.** is the final result concentration of the metabolite,

b) **LOD** is the *limit of detection* of the given metabolite,

c) $\mathbf{r}$ is the *raw concentration* i.e. the concentration equivalent of the resulting signal fit prior to comparing to **LOD** (relates to the blue area, cf. α)),

d) ρ is the correlation of lineshape metabolite signal with calculated fit characterizing the match between metabolite signal and fit (cf. β)),

e) $\boldsymbol{\Delta}$ is the concentration equivalent of the difference between metabolite signal and calculated fit (residue corresponding to the the red area, cf. γ)).

10.1.2 Different fit situations

Now we will describe the main fit cases.

i) In an ideal situation, where the fit corresponds fully to a metabolite signal well above **LOD**:

- the raw concentration is similar to the final result concentration,
- the correlation is $\geqslant$ 95% (indicated by ● displayed next to the value, otherwise the mark ○ is being used),
- the residue $\mathbf{\Delta}$ is close to zero mmol/L.

ii) Similar to situation described in i), but raw concentration below **LOD**. Generally, only an upper limit (e.g. $<$ **LOD**) can be provided. Especially, if the difference between raw concentration $\mathbf{r}$ and the final concentration **Conc.** is small, use the graphical figure displayed when pointing the cursor on the metabolite name for further visual inspection and validation. If a metabolite signal can be clearly discriminated from the rest of the spectrum and the calculated fit represents the respective signal well, the raw concentration may be used as approximative concentration estimate.

iii) Low correlation combined with large residual $\mathbf{\Delta}$. Such situation may arise in case of significant signal overlap, e.g. if a doublet signal of a metabolite to be quantified is overlaid with a large singlet. Use the graphical figure displayed when pointing the cursor on the metabolite name for further visual inspection and validation. If the non-overlaid part of the signal is well fitted, the final calculated concentration may still be used with confidence depending on the degree and nature of signal overlap.

iv) Combination of ii) and iii). Use the graphical figure displayed when pointing the cursor on the metabolite name for further visual inspection and validation.

10.2 Detailed definitions

Let s, f and b denote the functions describing the *raw spectra*, *fitted curve* and *(fitted) baseline* respectively. These functions are chosen such that $s \approx f + b$. Moreover, let I be a relevant PPM interval and P_N be the proton number for given metabolite/signal.

α) $\mathbf{r}$ (*raw concentration*) is defined as

$$\mathbf{r} = \frac{1}{P_N} \int_{\mathbb{R}} f(\xi)\,\mathrm{d}\xi.$$

β) ρ is the *correlation* of the functions s and $f + b$, i.e.

$$\rho = \max\bigl(0, \operatorname{corr}\bigl(\overline{s}, \overline{f + b}\bigr)\bigr)\,,$$

where $\overline{s}$, $\overline{f + b}$ are numerical representations of the functions s and $f + b$ on sufficiently fine mesh of the interval I.

γ) $\mathbf{\Delta}$ is the the area between the raw signal s and the fitted data $f+b$ on the interval I expressed in the term of the concentration, i.e.

$$\mathbf{\Delta} = \frac{1}{P_N} \int_I |s(\xi) - f(\xi) - b(\xi)|\,\mathrm{d}\xi.$$
